# Asynchronous development of *Zymoseptoria tritici* infection in wheat

**DOI:** 10.1016/j.fgb.2020.103504

**Published:** 2021-01

**Authors:** Elena Fantozzi, Sreedhar Kilaru, Sarah J. Gurr, Gero Steinberg

**Affiliations:** a School of Biosciences, University of Exeter, Exeter EX4 4QD, UK; b University of Utrecht, Padualaan 8, Utrecht 3584 CH, The Netherlands

## Abstract

The fungus *Zymoseptoria tritici* causes Septoria tritici blotch of wheat. Pathogenicity begins with spore germination, followed by stomata invasion by hyphae, mesophyll colonization and fruiting body formation. It was previously found that entry into the plant via stomata occurs in a non-synchronized way over several days, while later developmental steps, such as early and late fruiting body formation, were reported to follow each other in time. This suggests synchronization of the pathogen population *in planta* prior to sporulation. Here, we image a fluorescent *Z. tritici* IPO323-derived strain during infection. We describe 6 morphologically distinct developmental stages, and determine their abundance in infected leaves, with time post inoculation. This demonstrates that 3-5 stages co-exist in infected tissues at any given time. Thus, later stages of pathogen development also occur asynchronously amongst the population of infecting cells. This merits consideration when interpreting transcriptomics or proteomics data gathered from infected plants.

## Highlights

• *Zymoseptoria tritici* passes 6 morphologically defined stages during infection.
• Surface-located spores and hyphae are found for up to 17/18 days.
• Entry through stomata occurs from 1 to 13 days post infection.
• Mesophyll apoplast colonisation continuously increases during infection.
• Up to 5 stages co-exist in infected leaves at a given time.

## 1 Introduction

Septoria tritici blotch (STB) is one of the most devastating diseases of wheat, resulting in 5-10% loss of wheat yield *per annum* in the EU (Fones & Gurr, 2015). STB is caused by *Zymoseptoria tritici*. Under saprotrophic conditions, the fungus proliferates as multi-cellular spores. These asexual spores, as well as sexual ascospores, can infect wheat plants (Morais et al., 2015). Upon landing on the surface of a wheat leaf, they initiate pathogenic development by the formation of invasive hyphae, which enter the plant tissue via stomata (Duncan and Howard, 2000, Kema et al., 1996). This is followed by a biotrophic phase, during which the fungus colonizes the apoplastic space in the leaf mesophyll, without any obvious signs of plant damage (Deller et al., 2011, Kema et al., 1996, Steinberg, 2015). Upon reaching new stomata, *Z. tritici* hyphae form reproduction structures, the pycnidia, in substomatal cavities (Ponomarenko et al., 2011). These fruiting bodies mature and produce new macropycnidiospores, which are eventually released into the environment (Sackston, 1970; overview in Steinberg, 2015). This late phase of infection is accompanied by plant programmed cell death (Kema et al., 1996, Keon et al., 2007), thought to be initiated by the release of fungal necrosis factors (Kettles et al., 2017, M'Barek et al., 2015a, Motteram et al., 2009, Sanchez-Vallet et al., 2015).

During plant infection, *Z. tritici* goes through a series of developmental stages (Rudd et al., 2015). These steps are generally thought to be sequential, with germination of the spores on the leaf surface (0 -1 days post infection, =dpi), subsequent hyphal penetration of stomata (1-3 dpi), followed by mesophyll colonization (3-11 dpi) and pycnidia development in neighboring stomatal cavities (11 dpi +; Brennan et al., 2019). In particular, later during the infection, developmental stages, such as fruiting body formation and spore maturation) appear to follow each other in time, yet vary between different isolates of *Z. tritici* (Haueisen et al., 2019; see Fig. 3). However, recent work has shown that spores continuously germinate on the leaf, and hyphae penetrate stomata for up to 10 dpi (Fones et al., 2017, Haueisen et al., 2019). Thus, it was concluded that the early infection process is not synchronized (Seybold et al., 2020). Taken together, these data raise the possibility that the pathogen may synchronise during its development later in the infection process and prior to sporulation. Such process was described in the yeast *Saccharomyces cerevisiae* (Chia & van Werven, 2016). Alternatively, individual hyphae may follow their own developmental programme and result in non-synchronised infection during all developmental stages *in planta*.

In this brief study, we address this question using live cell imaging. We investigate the timing of wheat infection by the *Z. tritici* wildtype strain IPO323 (Kema & van Silfhout, 1997). Based on cell morphology and location in the plant, we define 6 developmental stages, beginning with spores and hyphae on the leaf surface, stomata penetrating hyphae, hyphae that colonize the apoplast, and early and maturing fruiting bodies. We use confocal laser scanning microscopy to quantify the occurrence of these stages in infected tissue over a period of 18 days. We show that infected plant material simultaneously contains *Z. tritici* structures in up to 5 developmental stages. Thus, invading hyphae are not synchronizing their pathogenic development, but co-exist in the same host tissue. Such asynchrony of the infection process merits consideration when drawing conclusions from data derived from transcriptomic and proteomic studies.

## 2 Results and Discussion

### 2.1 Defining developmental stages for analyzing *Z. tritici* infection

**Figure 1:**
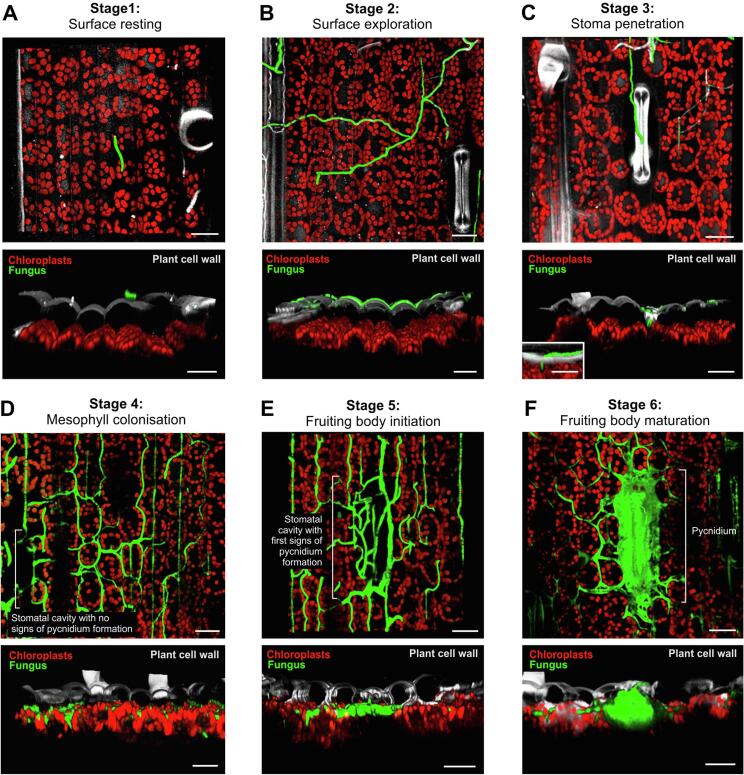
Infection stages of *Z. tritici* in wheat. (**A**) Stage 1, “Surface resting”. A “yeast-like” spore lands on the leaf surface, where it can survive for several days. During this time, it can switch to stage 2 and invade the plant. Note that this stage can last for up to 12 days, during which hyphal growth can be initiated (Fones et al., 2017). Plant epidermis (grey) and chloroplasts (red) are detected by their auto-fluorescence. Fungal cells express cytoplasmic eGFP (green). Upper image shows top view and lower image shows side view of a confocal image stack. Scale bars represent 20 µm. See Video 1. (**B**) Stage 2, “Surface exploration”. Spores can switch and form an infectious hypha, which is thin and grows directed. These hyphae explore the surface and eventually enter the leaf via stoma (Stage 3). Plant epidermis (grey) and chloroplasts (red) are detected by their auto-fluorescence. Fungal cells express cytoplasmic eGFP (green). Upper image shows top view and lower image shows side view of a confocal image stack. Scale bars represent 20 µm. See Video 1. (**C**) Stage 3, “Stoma penetration”. Hyphae enter the host through stomatal apertures. Plant epidermis (grey) and chloroplasts (red) are detected by their auto-fluorescence. Fungal cells express cytoplasmic eGFP (green). Upper image shows top view and lower image shows side view of a confocal image stack. Scale bars represent 20 µm. See Video 1. (**D**) Stage 4, “Mesophyll colonization”. The fungus grows in the apoplastic space between the intact cells of the mesophyll. During this “biotrophic” phase, no obvious infection symptoms are visible. Plant epidermis (grey) and chloroplasts (red) are detected by their auto-fluorescence. Fungal cells express cytoplasmic eGFP (green). Upper image shows top view (no images covering the epidermis was included) and lower image shows side view of a confocal image stack. Scale bars represent 20 µm. See Video 1. (**E**) Stage 5, “Fruiting body initiation”. Hyphae grow into the cavity of virgin stomata and begin to fill this space with fungal material. This developmental step marks the onset of the nectrotrophic phase, recognized by first signs of leaf chlorosis. Plant epidermis (grey) and chloroplasts (red) are detected by their auto-fluorescence. Fungal cells express cytoplasmic eGFP (green). Upper image shows top view (no images covering the epidermis was included) and lower image shows side view of a confocal image stack. Scale bars represent 20 µm. See Video 1. (**F**) Stage 6, “Fruiting body maturation”. Hyphae have filled the substomatal cavity and the fruiting body (pycnidium) begins to produce spores. Their number of spores per pycnidium was estimated to be 300 (Fones & Gurr, 2015). Plant epidermis (grey) and chloroplasts (red) are detected by their auto-fluorescence. Fungal cells express cytoplasmic eGFP (green). Upper image shows top view (no images covering the epidermis was included) and lower image shows side view of a confocal image stack. Scale bars represent 20 µm. See Video 1.

As outlined above, *Z. tritici* undergoes distinct morphological stages during its infection cycle. We used the eGFP-expressing strain IPO323_G (Kilaru et al., 2015) to follow the course of infection over time. We defined 6 microscopically distinct stages of fungal development during the infection of wheat leaves. At stage 1 (“Surface resting”; Fig. 1A, Video 1), single or multicellular spores have not yet switched to hyphal growth and appear to “rest” on the epidermis. Such un-germinated spores were reported to survive for up to 12 days on the surface of the leaf (Fones et al., 2017). These spores germinate (Ponomarenko et al., 2011) and explore the leaf surface as thin hyphae that grow directed (Stage 2 “Surface exploration”; Fig. 1B, Video 1), until they enter through the stomatal aperture (Stage 3, “Stoma invasion”; Fig. 1C, Video 1). This process is random (Fones et al., 2017) and multiple hyphae can enter the same stoma (Kema et al., 1996). Once inside the leaf, the invading hyphae colonize the apoplastic space, predominantly between the mesophyll and the epidermis (Stage 4, “Mesophyll colonisation”; Fig. 1D, Video 1). During this “biotrophic” phase, *Z. tritici* does not cause symptoms, yet it secretes plant cell wall-degrading enzymes and effectors (M'Barek et al., 2015b, Fantozzi et al., 2020). Upon reaching new stomata, hyphae grow to line the substomatal cavity (“stomata lining”; Duncan & Howard, 2000). This marks initiation of formation of the fruiting bodies (Stage 5, “Fruiting body initiation”; Fig. 1E, Video 1), but also coincides with the onset of the necrotrophic phase (Haueisen et al., 2019, Rudd et al., 2015), which is characterized by extended plant programmed cell death (Keon et al., 2007). Finally, new spores are formed that fill the substomatal cavity (Stage 6, “Fruiting body maturation”; Fig. 1F, Video 1). These mature pycnidia ultimately release these spores into the environment (Sackston, 1970).

### 2.2 Infected tissue contains *Z. tritici* structures in several different stages

After defining the developmental stages of *Z. tritici* IPO323_G during pathogenic development, we set out to determine their abundance relative to the time of first contact with the host. To this end, we acquired images randomly from infected tissue over a period of 18 days post infection (for more details see Material and Methods). Later time points were not included as (i) necrosis led to tissue auto-fluorescence and (ii) spore release from mature pycnidia compromised the analysis. We determined relative abundance every 24 h and categorizing them according to the infection stages defined in this study. In this way, a decrease in relative abundance of a given structure over time can be due to (i) the disappearance of structures in a certain stage (e.g. young fruiting bodies in stage 5 develop into mature pycnidia in stage 6) or (ii) an increase of fungal material in another stage (e.g. spores on the leaf surface may stay similar in absolute numbers, but their relative abundance decreases due to increased hyphal growth).

**Figure 2:**
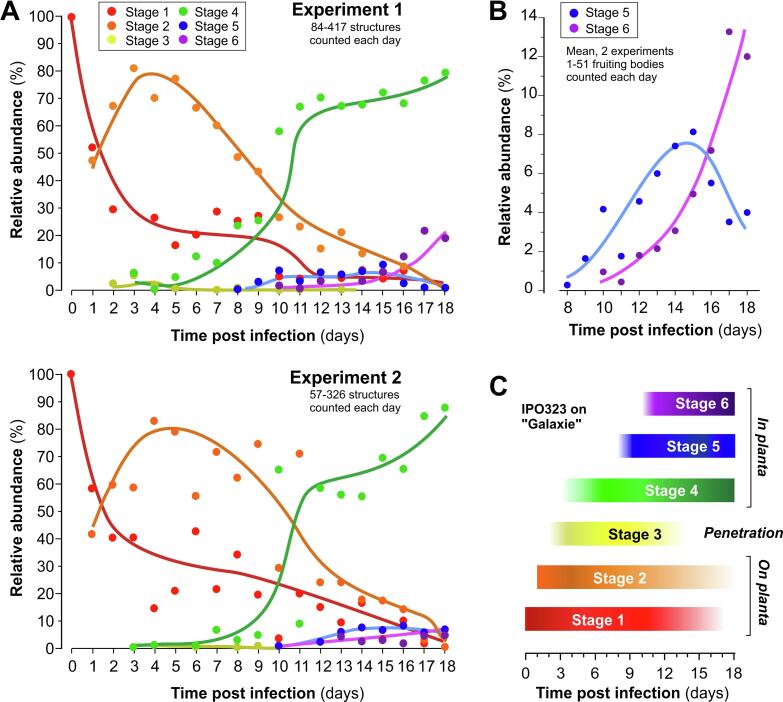
Relative abundance of the 6 developmental stages of the *Z. tritici* strain IPO323_G on and in infected wheat leaves for up to 18 dpi. (**A**) Graph showing the relative abundance of fungal structures at different days after infection in two independent experiments. Bread wheat leaves were infected with the cytoplasmic eGFP-expressing strain IPO323_G and microscopic images were taken. The sum of all structures was set to 100%. Regression curves were added manually. See Supplementary Table 1 for primary data. (**B**) Graph showing the relative abundance of early and late fruiting bodies (Stage 5 and 6) at 8-18 dpi. Data taken from data sets in (**A**). Regression curves were added manually. (**C**) Simplified graphical representation of the quantitative data shown in Fig. 2A.

In two independent experiments, we found that the various developmental stages overlapped significantly during the infection process (Fig. 2A, two independent experiments shown; note variation between both experiments; see Supplementary Table 1 for measured values). Consistent with Fones et al. (2017), spores and hyphae were found on the plant surface during most of the observation period (Stage 1: 0-17 days; Stage 2: 1-18 days; Fig. 2A, Supplementary Table 1). Hyphal penetration of stomata was observed from 2 dpi to 13 dpi, with a peak at day 3 and 4 (Stage 3, Fig. 2A, Supplementary Table 1). However, as infections can be caused by a single entry of a hypha (Fones et al., 2015), the number of hyphal penetration events seen was small. Mesophyll colonization was observed from day 3 onwards (Stage 4, Fig 2A, Supplementary Table 1). Initiation of fruiting bodies (=stomata cavity lining; Stage 5) began at 8 dpi and occurred until the end of the observation period (Fig. 2A, 2B, Supplementary Table 1). Finally, spore-containing pycnidia were observed at day 10 to 18 (Stage 6; Fig. 2A, 2B, Supplementary Table 1). These results demonstrate that 3-5 developmental stages co-exist temporarily in infected leaves during the initial 18 days of infection (Fig. 2C). It is important to note that these results are an indication of the relative abundance of structures in different stages of development at each day. These values are not indicative of cell numbers, as, for example, spores usually consist of 1-5 cells, whereas a mature fruiting body can contain ~300 of such spores (Fones & Gurr, 2015).

**Figure 3:**
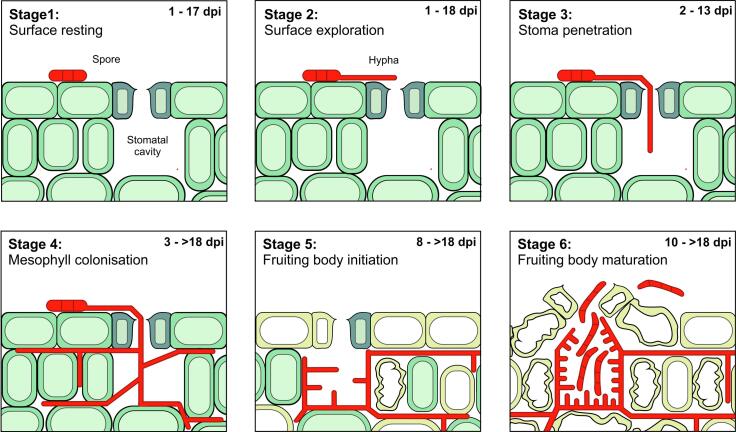
Overview of the timing of *Z. tritici* infection in wheat. The infection cycle can be described by a series of 6 stages. Individual hyphae go from one stage to the next, but as the population is not synchronized, up to 5 stages were found simultaneously in infected wheat leaves at a given time.

## 3 Conclusions

In this study, we define 6 developmental stages during the infection of wheat by *Z. tritici* which, from a cell biological perspective, differ from each other. In other words, a hypha that spreads within the mesophyll (Stage 4) differs from a hypha that increases branching activity and interacts with other hyphae to begin the formation of a fruiting body in the stomatal cavity (Stage 5). Several studies have addressed the infection cycle progression before. However, our quantitative analysis of these stages highlights that pathogenic development of *Z. tritici* cells is asynchronous amongst the population of infecting cells of the same strain. This is in particular obvious from the co-existence of early and late fruiting bodies in the same leaf sample (Fig. 2B), suggesting that even spore release from pycnidia is not synchronized. This finding contradicts reports that pathogenic development is reflected by developmental steps that can be related to days after infection (see Brennan et al., 2019). Instead, we consider it most likely that the asynchronous development of fruiting bodies reflects continuous germination and leaf penetration, which was reported to occur over up to 10 dpi (Fones et al., 2017, Haueisen et al., 2019). In this scenario, invading hyphae follow their own developmental program and may not be synchronised by the activity of a transcriptional regulator, such as Ime1p in budding yeast (Chia & van Werven, 2016).

Our molecular understanding of the host-pathogen interaction is largely based on transcriptomic and proteomic studies (Brunner et al., 2013, Haueisen et al., 2019, M'Barek et al., 2015a, M'Barek et al., 2015b, Mirzadi Gohari et al., 2015, Rudd et al., 2015, Yang et al., 2013, Yang et al., 2015). These studies used infected wheat leaves, harvested at defined time points after inoculation and the derived data were used to draw conclusions about the infection process. However, if a sample contains (i) surface-resting spores, (ii) surface-growing and (iii) stomata-invading, as well as (iv) mesophyll-colonising hyphae, the expression profiles will reflect the status of various conditions *in* and *on planta*. This may explain the finding that, for example, cutinases, which hydrolyze leaf cuticular waxes, are highly induced even at 4 days after contact of the fungus with the plant (Rudd et al., 2015). Such data may simply reflect the secretory activity of surface-growing hyphae, being the dominant stage at that time of infection. This limitation could be overcome by live cell imaging of gene expression *in planta*, using promoters fused to cytoplasmic GFP reporter constructs. Such an approach would complement the transcriptomic and proteomic approaches to interrogate the host/pathogen interaction.
